# Bibliographical Notices

**Published:** 1846-12

**Authors:** 


### PART III.—BIBLIOGRAPHICAL NOTICES.

### ARTICLE VII.

*Lectures on Natural and Difficult Parturition.* By Edward William Murphy, A. M., M. D., Prof. of Midwifery, University College, London: Obstetric physician, University College Hospital; and formerly assistant physician to the Dublin Lying-in Hospital. pp. 281. New York: S. S. & W. Wood. 1846. (From the Publishers.)

From a hasty glance over the pages of this work, we have arrived at the conclusion, that, although there are a great many works of this class extant, there is yet ample room for it. The arrangement of the subjects of the lectures, is excellent; maintaining the proper connexions, which are indispensable to a clear exposition.

We shall take occasion to refer to it hereafter; but in the meantime would recommend its perusal to students and physicians generally.

(For sale by Brautigam & Keen, Chicago.)

---

### ARTICLE VIII.

*New Periodical.* We have received the first number of "The Annalist," a record of practical medicine in the city of New York. Edited by Wm. C. Roberts, M. D. Containing 24 pages octavo; to be issued semi-monthly.

Judging from the contributors to this number, we should infer it to be under the especial patronage of the College of Physicians & Surgeons, and of course we shall expect it to be well conducted. The number before us bears evidence of much ability. We hope it will receive a liberal support, and welcome it to our list of exchanges.

